# Bioconversion of Meat and Fish-Based Former Foodstuffs by Black Soldier Fly Larvae: A Sustainable Pathway for Reducing Food Waste, Enhancing Nutrient Recovery, with a Circular Economy Approach

**DOI:** 10.3390/insects16050508

**Published:** 2025-05-09

**Authors:** Antonio Franco, Valentina Pucciarelli, Seyed Ali Hosseini, Eric Schmitt, Fulvia Bovera, Carmen Scieuzo, Patrizia Falabella

**Affiliations:** 1 Department of Basic and Applied Sciences, University of Basilicata, Via dell’Ateneo Lucano 10, 85100 Potenza, Italy; antonio.franco@unibas.it (A.F.); valentina.pucciarelli@unibas.it (V.P.); 2 Spinoff XFlies s.r.l, University of Basilicata, Via dell’Ateneo Lucano 10, 85100 Potenza, Italy; 3 Protix B.V., Industriestaat 3, NC 5107 Dongen, The Netherlands; seyedali.hosseini@protix.eu (S.A.H.); eric.schmitt@protix.eu (E.S.); 4 Department of Veterinary Medicine and Animal Production, University of Naples Federico II, Via Federico Delpino, 1, 80137 Napoli, Italy; bovera@unina.it

**Keywords:** bioconversion, meat and fish by-products, circular economy, food waste management, nutrient recovery

## Abstract

Food waste containing meat and fish presents a considerable environmental challenge due to regulatory constraints preventing its use in industrial insect farming. Although substrates derived from meat and fish are not currently approved for industrial insect feed production due to regulatory constraints, this study explores their potential in bioconversion through *Hermetia illucens* larvae. In this study, five different former foodstuffs containing meat and/or fish were tested to evaluate their suitability for BSFL rearing. The substrates included pizza with salami (PIZZA), cheeseburger (CHB), pasta Bolognese with meat (PASTA), chicken salad (CHISA), and fish salad (FISA). Results showed that BSFL successfully developed on all tested substrates. The highest performance was observed for FISA, with a total larval weight of 35.21 ± 3.91 g, dry matter yield of 11.21 ± 0.45 g, survival rate of 96.63 ± 0.40%**,** and the most efficient feed conversion ratio (FCR, 4.11 ± 0.59). Heavy metal analysis revealed substantial bioaccumulation of lead (Pb) and cadmium (Cd) in larvae. In particular, larvae reared on PIZZA showed a Pb concentration of 4.68 μg/100 g, with a corresponding bioaccumulation factor (BAF) of approximately 1.5. Cadmium accumulation was most notable in larvae fed CHB, with a Cd concentration of 0.41 ± 0.33 μg/100 g and a BAF of about 2.1. Despite this bioaccumulation, all detected concentrations remained well below the regulatory limits set by the European Union for animal feed, indicating not only the feasibility of *H. illucens* larvae in sustainable waste management but also its use as a safe protein source in animal feed. This research highlights the viability of integrating such food waste into insect bioconversion systems. With appropriate risk management, this practice could significantly improve nutrient recycling, waste management, and the circular economy, urging a regulatory review to allow broader substrate utilization. These positive outcomes underscore the potential of integrating currently restricted animal-derived food waste streams into *H. illucens*-based bioconversion systems, unlocking additional value for the circular economy and contributing to more efficient waste management practices.

## Simple Summary

Every year, large amounts of food waste containing meat and fish are discarded, creating serious environmental issues. At the moment, this type of waste cannot be used in industrial insect farming due to strict regulations. In this study, we tested whether food leftovers such as pizza, cheeseburgers, pasta with meat, chicken salad, and fish salad could be safely used to raise black soldier fly larvae (*Hermetia illucens*). The larvae grew well on all tested foods, especially on fish salad, showing excellent growth and conversion of food waste into biomass. We also checked whether the larvae accumulated harmful heavy metals such as lead and cadmium. Although some accumulation occurred, all values remained below the safety limits set by European regulations. This means that the larvae could still be used safely in animal feed. Our findings show that food waste containing meat and fish could be effectively recycled through insect farming. This approach could help reduce food waste and contribute to a more circular economy. However, current laws need to be updated to allow this environmentally friendly practice.

## 1. Introduction

Food waste represents a global issue with significant environmental, economic, and social consequences. The Food and Agriculture Organization (FAO) estimates that approximately one-third of all food produced for human consumption is wasted annually, amounting to about 1.3 billion tons. This not only entails the loss of valuable resources such as land, water, and energy but also significantly contributes to greenhouse gas emissions, particularly methane, which has a global warming potential 25 times higher than that of carbon dioxide. Additionally, the production of meat and fish requires substantial natural resources, including water and land. It is estimated that around 30% of the world’s agricultural land is used to produce food that ultimately goes to waste, contributing to resource inefficiency and waste accumulation [1,2]. Animal-derived food waste, such as meat and fish, presents an even greater environmental challenge, highlighting the need for innovative strategies to reduce waste and sustainably recover nutrients. In this context, the bioconversion of food waste through black soldier fly (*Hermetia illucens* L.) larvae (BSFL) emerges as an innovative and sustainable solution to address food waste issues. These larvae can significantly reduce waste mass and recover valuable nutrients, making the process both ecologically sustainable and economically advantageous [3,4]. BSFL are particularly effective at degrading a wide range of organic materials, converting them into high-value protein biomass and other useful products [5,6,7,8], with lower greenhouse gas emissions than traditional livestock systems [9]. However, the use of substrates containing meat and fish for BSFL farming presents specific challenges. Current European regulations prohibit the use of such substrates due to potential biological risks, including transmissible spongiform encephalopathies, prions, and other biological hazards [10,11,12]. Among these, *Escherichia coli* and *Salmonella* spp. are of particular concern, as they pose significant health risks to both animals and humans [13]. Some studies have shown that BSFL can reduce microbial loads of different pathogens in organic substrates, supporting the antimicrobial potential of BSFL [14,15,16,17,18]. Commission Regulation (EU) 2017/893 currently defines the types of substrates authorized for insect rearing for human and animal consumption, explicitly excluding meat and fish waste [12]. This regulation aims to safeguard public health, animal welfare, and food safety. However, these restrictions may limit the potential of insect farming to support a more sustainable global food system. Expanding the range of permissible substrates to include carefully managed and treated animal-derived waste could reduce environmental impacts and promote the principles of the circular economy by converting organic residues into valuable protein sources [19]. Heavy metal contamination in insect-based products is a critical concern. Heavy metals are persistent in the environment and may accumulate due to anthropogenic activities such as waste disposal, trophic transfer, and biomagnification. Some elements (e.g., selenium and zinc) are essential for organisms at low concentrations but become toxic when their levels exceed physiological thresholds. Others, such as mercury, arsenic, lead, and cadmium, can be toxic even at relatively low concentrations. Insects can accumulate these metals from contaminated substrates and feed [20,21,22], posing serious health risks to animals and humans through bioaccumulation and biomagnification along the food chain. Therefore, comprehensive monitoring of heavy metal levels is essential to ensure the safety and quality of insect-based feed. Additionally, by-products of insect rearing, particularly frass (a mixture of insect excrement and residual feed), should be evaluated concerning heavy metal presence due to their increasing use as organic fertilizer [23].

Although organic waste from fruits and vegetables and agricultural by-products is commonly recommended to ensure safety and quality, this study aims to explore whether substrates containing meat and fish could also be used safely when properly managed and treated. Research in this area may contribute to revising existing regulations and fostering more inclusive and sustainable practices. This article investigates the potential of BSFL to bioconvert food waste, including meat- and fish-based former foodstuffs (FFs), with particular attention to nutrient profiles and heavy metal content. By evaluating conversion efficiency and BSFL composition, the study aims to assess the feasibility of using these substrates as part of broader strategies to reduce organic animal-derived food waste and produce alternative protein sources.

## 2. Materials and Methods

### 2.1. Insect Rearing and Bioconversion Parameters

The experiment was conducted at the Protix Biosystems BV facility using 6-day-old (6-DOL) BSFL from the Protix BSF colony. Three hundred larvae were placed in each crate (food-grade plastic containers measuring 11 × 9 × 11 cm) and initially fed on a standard diet composed of processed wheat, for the first 6 days. After this period, the larvae were collected, washed, and counted. Then, 6-DOL were fed substrates made from former foodstuffs (FFs) containing meat or fish, including pizza with salami (PIZZA), cheeseburgers (CHB), pasta Bolognese with meat (PASTA), chicken salad (CHISA), and fish salad (FISA), purchased from supermarkets in the Netherlands and in Belgium. A detailed list of the ingredients used in each diet is reported in Table 1, while the macronutrient composition of each substrate is reported in Table 2. Since PASTA had a dry matter (DM) content below 22%, bread was added to bring the percentage up to this threshold. The DM of experimental diets ranged from 22% to 28%, and each experimental trial was set up to obtain the same feeding ratio (0.14 g (DM)/larvae). Throughout the experiment, the BSFL were kept in complete darkness at 28 °C and 65% relative humidity. At the end of the experiment, after 7 days of treatment, the larvae were separated from the residual material by manual sieving (mesh size 3 mm) to remove most of the adhering material. BSFL were then washed with distilled water and ethanol, stored at -20 °C, and then heated in a microwave (MAXINDUSTRIAL—Microwave Dryer MAXB-18B—Yantai, China) for further analysis.

The bioconversion parameters were calculated as follows:

$$Larval\;DM\;yield\;\left(\%\right)=Larval\;wet\;yield\;\left(g\right)\;\times \;Larval\;DM\;\times \;100$$

$$Survival\;rate\;(\%)=\frac{Number\;harvested\;larvae}{Number\;initial\;larvae}\times 100$$

$$FCR=\frac{Wet\;diet\;amount\;per\;cup\;(g)}{(Larval\;wet\;yield\;\left(g\right)-(6-DOL\;weight\;\left(g\right)\times 6-DOL\;DM))}$$

$$FCE=\frac{Larval\;wet\;yield\;\left(g\right)-6-DOL\;weight\;\left(g\right)\;}{Wet\;diet\;amount\;per\;cup\;(g)}$$

$$FCE\;(DM)=\frac{(Larval\;wet\;yield\;\left(g\right)\times larval\;DM)-(6-DOL\;weight\;\left(g\right)\times \;6-DOL\;DM)\;}{\left(Wet\;diet\;amount\;per\;cup\;\left(g\right)\;\times \;Diet\;DM\right)}$$

where *FCR* stands for feed conversion ratio, *FCE* stands for feed conversion efficiency, *DM* stands for dry matter, and 6-*DOL* refers to 6-day-old larvae.

### 2.2. Protein Contents of Larvae

To determine protein content, 10 g of larvae from each experimental trial were dried for 24 h at 55 °C in a Gallenkamp Hotbox Oven (Gallenkamp Labs, Cambridge, UK). The crude protein content was determined using the Kjeldahl method, according to AOAC [24], applying a nitrogen-to-crude protein conversion factor of 4.76, following Janssen et al. [25].

### 2.3. Lipid Extraction

The dried larvae from each experimental group were manually ground to a homogeneous biomass. Then, 5 g of the obtained biomass was weighed and placed into an extraction thimble within a Soxhlet extractor equipped with a condenser. Petroleum ether (150 mL) was used as the extraction solvent. The lipid extraction continued until the solvent inside the extractor became transparent (typically 25–30 cycles over 7–8 h) [26]. After the extraction, the solvent was removed using a rotary evaporator, and the obtained lipids were weighed. The lipid yield was calculated according to the following formula:

$$Lipid\;content\;(\%)=\frac{weight\;of\;lipids\;(g)}{weight\;of\;biomass\;(g)}\times 100$$

### 2.4. Fatty Acid Profile

The fatty acid profile of the extracted lipids was determined by GC-MS analysis. Lipids were first converted into their fatty acid methyl esters (FAME) derivatives using a base-catalyzed reaction, following Christie et al. and modified by Chouinard et al. methodologies [27,28]. FAMEs were analyzed using a FOCUS gas chromatograph (Thermo Fisher Scientific, Waltham, MA, USA) equipped with a fused silica SP^®^-2380 capillary column (100 m in length, 0.25 mm inner diameter, and 0.2 μm film thickness) (Supelco Inc., Bellefonte, PA, USA) and an AS 3000 II autosampler. Helium was used as the carrier gas, at a constant pressure of 180 kPa, with a split flow of 50 mL/min and an injection volume of 1 μL, following the guidelines of Zicarelli et al. [29]. Fatty acid peaks were identified by comparing retention times with commercial standards containing 37 FAMEs (Sigma-Aldrich Inc., St. Louis, MO, USA). The retention times of the conjugated linoleic acid (CLA) isomers were verified using commercial standards of these fatty acids (Larodan AB, Solna, Sweden). The area of each fatty acid was expressed as a percentage relative to the total area of the eluted peaks.

### 2.5. Ash Content of Larvae

Ash content was determined according to AOAC [24]. Briefly, around 2.5 g of sample were weighed into a porcelain crucible, dried in an electric oven at 103 °C until constant weight, and then incinerated at 550 °C overnight.

### 2.6. Mineral and Heavy Metals Profile Analysis

To analyze mineral and heavy metal content eventually occurring in initial substrates and consequently in larvae and the larval frass, samples (0.50 g ± 0.02) were placed in a Teflon vessel with 7.00 mL of 65% HNO_3_ and 3.0 mL of 30% H_2_O_2_ (Romil UpA). The vessel was sealed and subjected to a microwave-assisted digestion system (Milestone, Bergamo, Italy). Digestion was performed using a mineralization program for 50 min at 200 °C. After digestion, the vessel was cooled to 32 °C, and the resulting mixture was transferred to a 50.00 mL flask, with the final volume adjusted using Milli-Q water [30]. Subsequently, the mineralized samples underwent filtration using 45 mm SY25TF (PTFE) filters. To detect the concentrations of trace elements (using the ICP-OES technique), CETAC U5000AT (Thermo Fisher Scientific, Waltham, MA, USA) and Perkin Elmer Optima 2100 DV (PerkinElmer, Inc., Wellesley, MA, USA) instruments were employed. The purity of the chemicals was verified by running two blanks and a calibration curve during each analysis set [31]. For quality control, reference materials (BCR-422 cod muscle, IRMM Institute for Reference Materials and Measurements, DORM-2 fish protein, National Research Council, Ottawa, ON, Canada) were included, with results all within certified limits. The protocol from Perkin Elmer ICP application study number 57 was followed to determine the instrumental detection limits, which were expressed in wet weight [32]. Participation in interlaboratory studies organized by the Food Analysis Performance Assessment Scheme (Sand Hutton, UK) facilitated the performance assessment and reliability of the method.

### 2.7. Fiber Content of Larvae

To determine the fiber content,10 g of larvae from each experimental trial were dried for 24 h at 55 °C in a Precision Scientific Gallenkamp Hotbox Oven. Neutral detergent fiber (NDF) content was determined according to Van Soest et al. using an Ankom 220 Fiber Analyzer (Ankom Technology Corp., Fairport, NY, USA) [33]. Sodium sulfite was used in the NDF procedure, and both fractions were expressed as exclusive of residual ash.

### 2.8. Bioaccumulation Factor (BAF)

The bioaccumulation factor (BAF) was calculated to assess the ability of BSFL to bioaccumulate heavy metals from organic waste substrates. The *BAF* was calculated according to Walker [34] as follows:

$$BAF=\frac{concentration\;in\;larvae\;(Cf)}{concentration\;in\;initial\;substrate\;(Ci)}$$

where (Cf) is the concentration of the heavy metal in the larvae (μg/100 g DM) and (Ci) is the initial concentration of the heavy metal in the substrate (μg/100 g DM).

### 2.9. Statistical Analysis

All experiments were performed in biological triplicates. Results are expressed as means ± standard deviation. Data normality was assessed using the Shapiro-Wilks test. Data were analyzed with one-way ANOVA (analysis of variance) and Tukey’s *post hoc* tests. All statistical analyses were performed using GraphPad Prism version 6.01 for Windows (GraphPad Software, La Jolla, CA, USA; www.graphpad.com (accessed on 2 April 2025)).

## 3. Results and Discussions

### 3.1. Bioconversion Data

This study aimed to evaluate the effects of different former foodstuffs (FFs) containing meat and fish—PIZZA, CHB, PASTA, CHISA, and FISA—on the growth, feed conversion efficiency (FCE), and substrate utilization of BSFL. Significant differences were observed in larval weight, survival, substrate reduction, and FCE across the tested substrates. These results were analyzed and compared with existing literature.

To obtain the aforementioned parameters, BSFL growth and weight were monitored both at the beginning and the end of the trial, along with feeding substrate consumption. In particular, before starting the experiment, the weight and DM content of 6-day-old larvae were measured. The average weight of a single larva was 7.46 ± 1.80 mg, and the larval DM content was 33%. The bioconversion data for larvae reared on different FF diets are presented in Table 3.

The nutritional data in Table 1 and Table 2 provides a detailed breakdown of various experimental diets used for BSFL rearing. These diets vary significantly in their protein, fat, carbohydrate, and fiber content, all of which have been shown to influence larval growth, body composition, and FCE. Based on literature, specific predictions can be made regarding how these diets might affect key life-history traits in BSFL.

Total larval weight was highest in larvae fed FISA (35.21 ± 3.91 g), followed by CHISA (32.56 ± 4.66 g) and PIZZA (32.28 ± 11.24 g). CHB yielded the lowest total larval weight (24.52 ± 8.34 g), indicating reduced growth efficiency on this substrate. Regarding the survival rate, FISA had the highest survival (96.63 ± 0.40%), suggesting its high suitability for larval rearing. PIZZA showed a relatively high survival rate (80.18 ± 18.25%), whereas CHB resulted in the lowest (70.25 ± 18.02%), suggesting possible nutritional inadequacies or low palatability of the substrate. The feed conversion ratio (FCR) and feed conversion efficiency (FCE) are complementary indicators used to assess how effectively larvae convert feed into biomass. A lower FCR value indicates better efficiency, while higher FCE values reflect better conversion. As shown in several studies, including those of Barragan-Fonseca et al. and Cammack & Tomberlin, carbohydrate-rich diets, such as PIZZA and CHB (22 g and 20 g of carbohydrates, respectively), can enhance FCE [35,36], as carbohydrates serve as a readily accessible energy source for faster growth. However, while the CHB diet provided a relatively balanced macronutrient profile (13.00 g of protein, 20.00 g of carbohydrates), its high fat content (11.00 g) may have contributed to reduced efficiency, as excess fat is more likely to be stored rather than converted into biomass [35]. In contrast, the PASTA diet (4.50 g of protein, 15.00 g of carbohydrates), though lower in both protein and fat, may have led to favorable FCR and FCE values by enhancing biomass conversion efficiency, potentially at the expense of larval protein accumulation. Among all tested diets, FISA resulted in the most efficient feed utilization, as indicated by the lowest FCR value (4.11 ± 0.59), followed by CHISA (4.82 ± 0.94) and PIZZA (5.44 ± 2.29). The highest FCR, indicating the least efficient conversion, was observed in larvae fed CHB (7.34 ± 2.70). FCE, being the reciprocal of FCR, offers complementary insight into feed utilization. FISA showed the highest FCE-DM value (0.25 ± 0.00), confirming its superior conversion efficiency, while CHB had the lowest FCE and FCE-DM values (0.15 ± 0.05 and 0.15 ± 0.06, respectively). CHISA and PIZZA also performed well, with FCE-DM values of 0.22 ± 0.05. In conclusion, FISA emerged as the most efficient substrate in terms of total larval weight, DM weight, survival rate, and FCR/FCE values. In contrast, CHB consistently performed the worst, underlining poor conversion efficiency and feed utilization. The relationship between macronutrient composition and conversion performance aligns with prior studies, such as Barragan-Fonseca et al., who emphasized the nutritional plasticity of BSFL [37]. According to Table 2, the CHB diet (13.00 g of protein, 20.00 g of carbohydrates) provides a balanced macronutrient profile, while the PASTA diet (4.50 g of protein, 15.00 g of carbohydrates) is more carbohydrate-based. This protein–carbohydrate balance is crucial for both larval growth and body composition. High-protein diets, such as the CHB and pizza, tend to promote faster larval growth [36] but may also increase fat storage in larvae [37]. On the other hand, lower-protein diets may slow larval growth and reduce body fat accumulation while improving FCR, consistent with observations for the PASTA diet. Larval body composition, particularly the accumulation of fat and protein, is heavily influenced by dietary macronutrients. According to Beniers & Graham, high-protein diets, such as the CHB, generally increase body protein content in larvae [38], while balanced diets, such as FISA (5.40 g of protein and 4.30 g of fat), promote moderate fat accumulation without compromising protein synthesis. Fuso et al. also demonstrated that balanced nutrient intake ensures a more stable body composition, with a healthy fat-to-protein ratio [39]. In conclusion, the FISA diet (5.40 g of protein and 4.30 g of fat) appears to be the most efficient substrate across all bioconversion metrics, combining favorable larval growth, survival, and nutrient profile with the best FCR and FCE values. CHB, in contrast, consistently showed poor conversion outcomes, highlighting the critical role of macronutrient balance in optimizing BSFL performance.

Although fiber content in the diets ranged from 0.90 to 2.10 g, it did not significantly affect bioconversion performance or larval development in this study. This suggests that, within the tested range, fiber may not be a limiting factor. While previous studies have shown that high-fiber substrates may require nitrogen supplementation to support growth [40], further research would be needed to clarify its role under different conditions.

In the context of the diets listed in the table, FISA (1.97 g of fiber) and CHB (1.30 g of fiber) have relatively higher fiber levels. While this could enhance substrate digestibility, leading to more efficient nutrient uptake and growth, caution is necessary. Excess fiber, as highlighted by Hopkins et al. [40] and Palma et al. [41] may reduce growth rates and conversion efficiency. Therefore, balancing fiber content, particularly in nitrogen-poor feedstocks, is critical for maintaining efficient larval bioconversion [40,41].

Salt content in diets may also influence larval performances, although research on *H. illucens* larvae and salt tolerance remains limited. CHB had the highest salt content among the diets listed (1.4 g), which could potentially affect larval development by altering osmotic balance. According to Li et al., larvae performed optimally with a salt concentration between 0.5% and 2%, leading to higher larval weight and lipid accumulation [42]. However, growth and survival rates declined up to 50% in salinities above 4% due to the increased energy cost for osmotic regulation and reduced nutrient absorption efficiency. Despite CHB having the highest salt level, the sodium content measured in larvae was not the highest (as observed in larvae fed FISA), suggesting that sodium assimilation and retention depend on additional factors beyond dietary salt concentration.

The findings of the present study demonstrate that the composition of animal-derived food waste substrates significantly influences the growth, FCE, and survival of *H. illucens* larvae, with FISA identified as the most effective substrate in terms of bioconversion efficiency. Larvae reared on FISA exhibited higher total larval weight, DM content, and survival rates than those fed other substrates, including PIZZA and CHB. These outcomes align with previous research by Chaklader et al., who demonstrated that protein-rich substrates derived from fish waste enhance larval performance due to high protein availability, improving nutrient conversion and biomass accumulation [43]. This supports the idea that nutrient-dense and protein-rich substrates, such as fish-based waste, optimize larval development and conversion efficiency. In contrast, CHB resulted in lower larval weight, DM content, and survival, reflecting poor feed conversion performance. This may be due to its high fat and processed content, which could negatively affect larval metabolism. Similar observations were reported by Kawasaki et al., who found that diets high in fat content reduced *H. illucens* larval growth and increased mortality [44]. The higher FCR value observed in larvae fed CHB (7.34 ± 2.70) supports this interpretation, as more feed was required to produce less biomass. Survival also varied significantly among diets: FISA showed the highest survival rate (96.63 ± 0.40%), while CHB had the lowest (70.25 ± 18.02%).- These findings are consistent with previous observations by Nguyen et al. [45], which emphasized the importance of resource quality in determining larval viability. In this context, the nutritional complexity and processing level of the substrate could affect larval development outcomes. These results suggest that some processed animal-derived food waste may require supplementation or refinement before being used efficiently in larval production. Nonetheless, even suboptimal substrates could still be valuable for waste management, given the larval ability to convert diverse organic material. Regarding FCE, FISA and CHISA had the most favorable values (0.17 ± 0.02 and 0.18 ± 0.03, respectively), while CHB again showed the lowest performance (0.15 ± 0.05). These findings underscore the importance of substrate composition in determining how efficiently *H. illucens* converts feed into biomass. Balanced, protein-rich diets are more effectively utilized, highlighting the need for careful substrate selection in insect bioconversion systems. One limitation of the present study is the variability in composition among the animal-derived food waste substrates used, which may have influenced the results. Kalová & Borkovcová also highlighted that consistency in substrate composition is critical for achieving optimal larval growth and FCE [46]. In conclusion, different feeding substrates provide varying macronutrient profiles that affect larval performances. Diets high in both protein and carbohydrates, such as CHB, may support rapid growth but also promote fat accumulation, while low-protein diets like PASTA may slow growth but improve FCR, depending on production goals. Therefore, diet should be tailored to specific objectives, whether to maximize total biomass, increase larval protein content, or optimize conversion efficiency.

Future studies should focus on standardizing substrate composition or developing blended food waste streams to reduce variability and enhance bioconversion outcomes.

### 3.2. Larval Composition

The analysis of the nutritional composition of BSFL reared on different substrates revealed significant variations across all measured parameters, including moisture, crude protein, ether extract, ashes, and crude fiber.

The nutritional value of larvae reared on different FF diets is presented in Table 4, Table 5 and Table 6.

Larvae reared on the PIZZA substrate exhibited the highest moisture content (29.648 ± 1.179%), significantly higher than CHB (26.585 ± 1.174%), while the other substrates—PASTA, CHISA, and FISA—showed comparable moisture values. Regarding crude protein, larvae reared on CHB had the highest concentration (43.761 ± 0.789%), significantly greater than those reared on PASTA (40.076 ± 0.966%) and CHISA (40.879 ± 2.380%). Protein levels in PIZZA and FISA (41.628 ± 2.564% and 43.236 ± 1.543%, respectively) were comparable to CHB. In terms of fat content, CHB also showed the highest value (25.510 ± 1.473%), significantly higher than PIZZA (20.240 ± 2.553%), while PASTA, CHISA, and FISA exhibited similar fat yields. Ash content was highest in larvae fed PIZZA (4.110 ± 1.042%), while CHB, PASTA, CHISA, and FISA showed lower and more uniform values. As for crude fiber, larvae reared on PIZZA showed the highest content (4.375 ± 0.848%), followed by PASTA, CHISA, and FISA, with CHB showing the lowest content (1.519 ± 0.421%). Mineral profile analysis revealed considerable variability among the groups. Calcium levels peaked in larvae fed PIZZA (240.365 ± 25.555%), whereas phosphorus and potassium were highest in FISA (311.635 ± 31.043% and 438.700 ± 91.724%, respectively). PASTA and FISA had the highest magnesium content (62.860 ± 6.584% and 56.677 ± 7.287%). Zinc and iron were most abundant in CHB and FISA; the latter was also rich in sodium and copper. Manganese content peaked in PIZZA (0.450 ± 0.063%), while the highest selenium levels were detected in larvae fed FISA (48.948 ± 10.274%).

The comparison between the FF nutrient composition and the larvae reveals interesting correlations. Larvae fed CHB and FISA exhibited similarly high protein levels (43.761 ± 0.789% and 43.236 ± 1.543%, respectively), despite the notable difference in protein content of their respective diets (13.0 ± 1.5 g for CHB and 5.4 ± 3.2 g for FISA). This suggests larval efficiency in converting available protein from substrates into biomass, even when the protein availability is low. Indeed, despite the PIZZA substrate having a lower protein content (10.01 ± 1.55 g), the resulting larvae resulted in a relatively high protein concentration (41.628 ± 2.564%), suggesting high protein conversion efficiency. Lu et al. and Seyedalmoosavi et al. similarly emphasize that BSF larvae are highly capable of turning low-protein organic waste into a high-protein biomass, between 41% and 54% of DM [47,48]. This efficiency is a clear demonstration of the larvae’s ability to process and accumulate protein beyond what might be expected based on the initial composition of the diet, reflecting their adaptability and efficiency in nutrient absorption. Barragan-Fonseca et al. also noted that the protein content in larvae remains relatively stable, especially when diets are not highly enriched with proteins, supporting our results [49]. This may be attributed to the expression of specific digestive enzymes, like proteases, which facilitate the extraction and assimilation of amino acids from various sources, even those lower in protein content [35,50]. Some studies suggest a compensatory metabolic mechanism allowing larvae to maintain high protein accumulation under suboptimal dietary conditions [50]. As concerns lipids, larvae fed CHB (25.510 ± 1.473%) showed significantly higher lipid accumulation compared to those fed other diets, reflecting the higher fat content of the CHB diet (11.0 ± 2.3 g). Interestingly, despite the lower fat content of the FISA diet (4.3 ± 1.9 g), larvae accumulated a considerable amount of lipids (23.314 ± 1.518%), suggesting that fat assimilation is influenced by additional factors other than dietary fat availability. Similarly, larvae fed PIZZA, despite the relatively high fat content of this diet (12.0 ± 2.4 g), showed only moderate lipid levels (20.240 ± 2.553%), further suggesting a non-linear relationship between dietary fat and larval lipid content.

Examining lipid accumulation, our results show that larvae fed CHB (11.0 ± 2.3 g of dietary fat) accumulated high levels of ether extract (25.510 ± 1.473%). This result aligns with findings by Seyedalmoosavi et al. and Lu et al., who observed that BSF larvae can accumulate significant amounts of lipids, ranging from 11.8% to over 40% of DM, depending on the fat content of the substrate [47,48]. Barragan-Fonseca et al. also found that the larval crude fat concentration is highly influenced by the nutrient profile of their diet, which matches our findings of elevated lipid content in larvae fed fat-rich diets [37]. Lipid metabolism is regulated by enzymatic mechanisms, such as lipase and other fat-digesting enzyme expression and activity, allowing for the storage of energy reserves in response to dietary availability [51,52]. These lipid storage mechanisms are particularly advantageous in environments where nutritional quality may fluctuate, such as organic waste, a common substrate for rearing *H. illucens* larvae.

Crude fiber content in larvae generally reflected the dietary fiber level: diets with low fiber content, such as CHB (1.3 ± 0.2 g) and CHISA (0.9 ± 0.4 g), resulted in lower crude fiber content in larvae (1.519 ± 0.421% and 2.682 ± 0.782%, respectively). However, despite the PIZZA diet having a similar fiber content to CHB (1.3 ± 0.3 g), larvae fed PIZZA showed significantly higher fiber levels (4.375 ± 0.848%). This suggests that other factors, such as digestibility or substrate matrix complexity, may affect fiber accumulation in larvae.

Previous studies by Barragan-Fonseca et al. and Lu et al. highlighted that dietary fiber impacts both larval growth and digestibility, as fiber-rich diets require more energy for digestion and processing [37,47].

Mineral assimilation in the larvae, indicated by ash content, was highest in larvae fed PIZZA (4.110 ± 1.042%), reflecting the relatively higher mineral content in this diet. Lu et al. and Seyedalmoosavi et al. observed that BSF larvae were capable of accumulating significant amounts of minerals like calcium, phosphorus, and potassium, depending on the availability in their diet [47,48]. The high mineral content in larvae fed PIZZA could be linked to its higher carbohydrate and protein content, which contributes to the retention of essential minerals. The study by Barragan-Fonseca et al. further supports this, noting that dietary mineral content is crucial for larval development and significantly influences their ash composition [37]. This concept highlights the larvae’s ability to thrive on diverse substrates with varying nutrient profiles, efficiently converting them into high-quality protein, lipids, and other essential nutrients. Overall, the obtained results reinforce the nutritional plasticity of BSF. Despite differences in substrate composition, larvae effectively adjusted their nutrient uptake and body composition, maintaining relatively stable protein and lipid levels. This adaptability makes BSF larvae a promising waste-to-nutrient conversion system, capable of turning low-value organic waste into valuable biomass for animal feed and other applications.

### 3.3. Heavy Metals Concentration in Initial Substrates, Larvae, and Larval Frass

**Figure 1:**
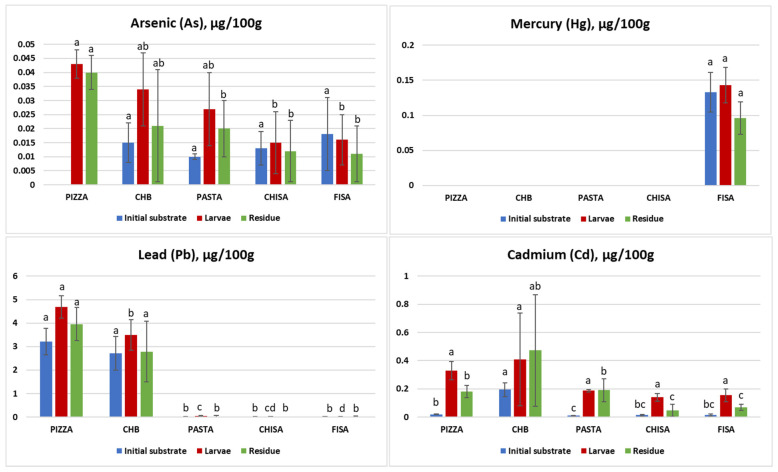
This figure shows the concentrations in μg/100 g of arsenic (As), mercury (Hg), lead (Pb), and cadmium (Cd) in initial substrates (blue), larvae (red), and residue (frass) (green) for various feeding substrates: pizza with salami (PIZZA), cheeseburger (CHB), PASTA, chicken salad (CHISA), and fish salad (FISA). The comparison highlights differences in heavy metal content across the three matrices. Data are expressed as means ± standard deviation from three independent biological replicates. Statistical analysis was performed with one-way ANOVA and Tukey’s *post hoc* test. Different letters within the same diet indicate significant differences among sample types (initial substrate, larvae, and residue).

The concentrations of arsenic (As), mercury (Hg), lead (Pb), and cadmium (Cd) were measured in the initial substrates, BSFL and frass, after rearing. The substrates used included PIZZA, CHB, PASTA, CHISA, and FISA. Data are reported in Figure 1 and Supplementary Table S1.

### 3.4. Bioaccumulation Factor (BAF)

The bioaccumulation factors (BAF) for various heavy metals in *H. illucens* larvae reared on different substrates revealed significant metal uptake.

Results are reported in Table 7. A literature comparison is provided in Table 8.

The comparison of the heavy metal concentrations among the three sample categories (initial substrates, larvae, and residue) reveals that BSFL effectively bioaccumulate metals from their respective substrates. In particular, Pb and Cd showed significant accumulation in larvae across various substrates. As and Hg, while detected in lower concentrations, also exhibited notable bioaccumulation, particularly from substrates with initially higher concentrations (e.g., fish salad for Hg). These findings align with existing literature and underscore the need for and the importance of stringent control measures to ensure the safety of insect-based products intended for animal feed or human consumption. According to the European Union Commission Regulation (EC) No 629/2008, the maximum permissible levels for heavy metals in feed are: As 2 mg/kg, Cd 1 mg/kg, Pb 10 mg/kg, and Hg 0.1 mg/kg. The concentrations observed in this study are well below these regulatory limits, indicating that, with appropriate substrate selection and monitoring, the risk of heavy metal contamination can be effectively managed. The minimal accumulation of As and Hg is a positive outcome, further supporting that with proper substrate selection, the risk to food safety remains low. However, the significant bioaccumulation of Pb and Cd, although within permissible limits, highlights the need for ongoing vigilance and control in insect farming practices.

Additionally, contaminants in frass, the by-product of insect rearing, could present potential hazards when used as fertilizer. Therefore, it is essential to detect and quantify heavy metals in frass to ensure its safe use in agriculture. Our analysis indicates that the concentrations of heavy metals in frass were very low, suggesting that it does not pose a significant risk to soil health or plant growth when used as fertilizer. This finding supports the safe agricultural application of frass, provided contaminant levels are regularly monitored and maintained within safe limits.

#### 3.4.1. Arsenic (As) Accumulation

Arsenic bioaccumulation has been documented even when its concentration in the rearing substrate was below detectable limits (as in the PIZZA substrate, in which no As was detected), suggesting that the larvae may gradually accumulate trace amounts over time due to their feeding and metabolic processes. Our data show an As very low accumulation (0.043 ± 0.005 μg/100 g), even though As was not detected in the initial substrate. The presence of As in larvae despite its undetectable levels in the substrate could also result from differences in analytical detection limits, typically lower in biological samples than in complex organic matrices, allowing BSFL to gradually accumulate As traces not initially identified in the substrate. Indeed, analytical methods often have lower detection limits (LOD) for biological samples than for complex organic waste matrices, meaning that As ultra-trace levels in the substrate may be missed but still bioaccumulated in larvae tissues over time [31]. Additionally, BSFL continuously ingest and metabolize large amounts of feed substrate, allowing for gradual accumulation of trace metals [53]. Studies indicate that BAF values for As in BSFL range between 0.49 and 0.58 when larvae are reared on substrates containing As concentrations of 4 to 16 mg/kg, implying a moderate bioaccumulation potential [53]. This phenomenon may reflect larval capacity for selective metal absorption and retention, possibly influenced by gut microbiota interactions affecting metal speciation and sequestration [31]. Additionally, Lievens et al. reported that As concentrations in BSFL decline after a starvation period, suggesting that part of the detected As remains within the gut content rather than being systemically integrated into larval tissues [54].

#### 3.4.2. Mercury (Hg) Accumulation

Mercury accumulation was observed exclusively in larvae reared on fish salad (0.143 ± 0.025 μg/100 g). This finding aligns with Purschke et al., who documented similar low Hg bioaccumulation in larvae reared on fish-based substrates [55]. The reduction in Hg levels in residues indicates a significant uptake by larvae, supporting the idea of BSFL efficiency in sequestering Hg from diet. For Hg, the BAF of larvae reared on fish salad was approximately 1.1 (0.143 μg/100 g in larvae vs. 0.133 μg/100 g in substrate). This is further supported by Biancarosa et al., who observed comparable Hg bioaccumulation in BSFL fed with contaminated substrates, reporting BAFs from 1.0 to 1.2 in larvae fed seaweed-enriched substrates [56]. Conversely, Purschke et al. documented Hg BAF lower than 1, suggesting the larvae’s lower efficiency in bioaccumulating Hg compared to other metals [55].

#### 3.4.3. Lead (Pb) Accumulation

Lead showed high BAFs across several substrates. The comparative analysis with residues shows a metal uptake and retention pattern consistent with other research indicating that larvae can effectively bioaccumulate and retain Pb from feed. This is supported by Van der Fels-Klerx et al., which reported BAF values for Pb between 1.2 and 1.8 in larvae reared on contaminated substrates [57]. In contrast, Diener et al. observed Pb BAF values up to 1.0 in similar rearing conditions, with no significant accumulation among BSFL samples [58].

#### 3.4.4. Cadmium (Cd) Accumulation

Cadmium was detected in all substrates, with the highest concentration in CHISA (0.195 ± 0.049 μg/100 g), leading to a notable accumulation in larvae (0.408 ± 0.329 μg/100 g), resulting in a BAF of approximately 2.1. Diener et al. reported similar Cd accumulation trends in BSFL (up to 2.2), especially when reared on substrates with higher initial Cd concentrations [58]. The consistency of Cd levels in residues further supports the hypothesis that BSFL can effectively sequester and concentrate Cd from contaminated substrates. This is corroborated by Charlton et al., who detected Cd accumulation in larvae fed with organic waste [59]. Purschke et al. observed Cd BAFs of 9.1, higher than other studies in larvae fed contaminated feed, highlighting the strong bioaccumulation potential [55].

Taken together, both our results and literature data indicate that Cd is the most bioaccumulated heavy metal. Nonetheless, the Cd level in our study remained below the limits set by EC Regulation Directive 2002/32/EC, as amended for heavy metals by Regulation (EU) 2015/186.

**Table 8:** Table showing the concentration of various heavy metals (arsenic (As), mercury (Hg), lead (Pb), and cadmium (Cd)) in different initial substrates and the corresponding concentration detected in larvae after consumption. Data are presented from multiple studies, including both contaminated and non-contaminated substrates, to illustrate the range of BAF values observed under different conditions.

References	Initial Substrate	Heavy Metal	Concentration in Substrate (μg/100 g)	Concentration in Larvae (μg/100 g)	BAF
Present study	Fish salad	Hg	0.133	0.143 ± 0.025	1.1
Purschke et al. [55]	Fish-based substrates	Hg	0.2	0.1	<1
Biancarosa et al. [56]	Contaminated substrates	Hg	0.1–0.4	0.1–0.5	1.0–1.2
Present study	Pizza with salami	Pb	3.22 ± 0.563	4.678 ± 0.478	~1.5
Van der Fels-Klerx et al. [57]	Contaminated substrates	Pb	0.5–1.5	0.6–2.7	1.2–1.4
Diener et al. [58]	Lead-contaminated substrates	Pb	1–5	1–5	1.0
Tschirner and Simon [50]	Plant-based substrates	Pb	0.24–0.86	0.55–2.68	2.6
Purschke et al. [55]	Contaminated feed	Pb	15.2	35.6	>2
Present study	Cheeseburgers	Cd	0.195 ± 0.049	0.408 ± 0.329	~2.1
Van der Fels-Klerx et al. [57]	Cadmium-contaminated substrates	Cd	0.1–1.5	0.15–3.75	6.1–9.5
Diener et al. [58]	Contaminated substrates	Cd	1–10	2–30	2.32–2.94
Purschke et al. [55]	Contaminated feed	Cd	1.5	13.7	9.1
Tschirner and Simon [50]	Plant-based substrates	Cd	0.09–0.23	0.47–2-24	7.4
Wu et al. [60]	Contaminated substrates	Cd	-	-	0.1–0.32
Gao et al. [61]	Contaminated substrates	Cd	-	-	4.63

## 4. Conclusions

This study highlights the remarkable efficiency and nutritional plasticity of *Hermetia illucens* larvae in converting diverse substrates, including those containing animal by-products, into protein- and fat-rich biomass. Our findings align with existing literature, demonstrating that BSFL are highly effective at assimilating nutrients from a variety of organic waste sources, including fish- and meat-based substrates. This adaptability underscores the potential of BSFL as a sustainable and scalable solution for biomass production in the feed and bio-based industries. The results also emphasize the importance of reconsidering and re-evaluating current regulations that restrict BSFL rearing to plant-based substrates [10,11]. A growing body of evidence suggests that, with proper hygiene controls and treatment protocols, substrates containing meat and fish can be utilized safely and effectively. Although no plant-based control was included in the present study, the tested animal-based substrates demonstrated excellent performance within their group. Broadening the range of permissible substrates would not only enhance the nutritional yield of larvae but also contribute significantly to circular economy models by transforming underutilized animal-derived food waste into valuable, nutrient-dense biomass. The ability of BSFL to efficiently convert animal-based substrates into high-quality protein and fat offers a strategic opportunity to optimize their use in industries such as aquaculture, livestock feed, and biofuel production. Furthermore, the potential to fine-tune larval nutrient profiles by adjusting diet composition presents an opportunity to tailor production to meet specific market demands. Moreover, this study highlights the importance of monitoring heavy metal contamination in food waste substrates used for BSFL rearing. While substantial bioaccumulation of Pb and Cd in larvae was observed, the accumulation of As and Hg remained relatively low across most substrates, demonstrating the minimal accumulation of these metals. Low BAF values for As and Hg suggest that BSFL may not significantly concentrate these metals, especially when present at low concentrations in the substrates. This is particularly relevant for substrates with negligible or undetectable levels of As and Hg, where the larvae did not show appreciable bioaccumulation. From a regulatory perspective, updating policies to include animal-based substrates, supported by rigorous safety protocols, would ensure both the safety and efficiency of bioconversion processes. Such changes could unlock new economic and environmental benefits, enhancing the role of *H. illucens* in sustainable waste management and nutrient recycling. As scientific understanding advances, regulatory evolution should evolve accordingly, facilitating the safe and responsible use of meat- and fish-based substrates in future sustainable food and feed systems. These findings contribute to the growing body of evidence supporting the safe and effective use of animal-based substrates in BSFL rearing.

## Figures and Tables

**Table 1:** Ingredient composition of experimental diets used for *Hermetia illucens* larvae rearing (as reported on the product label). Legend: ✓ = ingredient included; percentage = inclusion level; empty cell = ingredient not included.

INGREDIENT (% Inclusion)	PIZZA	CHEESEBURGER	PASTA	CHICKEN SALAD	FISH SALAD
Wheat flour	✓	✓		✓	
Vegetables	24.20%		5.20%		
Milk	✓	✓		✓	
Pork	12.00%				
Beef	5.70%	43.00%	10.00%		
Starch	✓				
Oil	✓			✓	
Herbs	✓		✓		
Yeast	✓	✓			
Onion		✓			
Vinegar		0.80%			
Soy protein		✓			
Spices			✓	✓	
Romaine Lettuce				18.00%	
Iceberg lettuce				18.00%	
Chicken breast				14.00%	
Egg				8.80%	
Potato				✓	25.00%
Potato starch		✓			
Mackerel					18.00%
Bread					18.00%
Durum wheat			✓		
Tomato			21.00%		
Tomato concentrate		2.10%			
Tomato paste			2.40%		
Rapeseed oil			✓		
Corn starch			✓		
Lettuce					39.00%

**Table 2:** Nutritional composition of the experimental diets used for rearing *Hermetia illucens* larvae, based on values declared on the product labels. The diets differ in DM content (%), energy (kcal), and macronutrient composition, including protein, fat, carbohydrates, fiber, sugar, and salt. Nutrient levels are presented in grams per 100 g of DM.

ID	Diet DM (%)	Energy (kcal)	Protein (g)	Lipids (g)	Sugar (g)	Fiber (g)	Carbs (g)	Salt (g)
PIZZA	28.00	239.00	10.00	12.00	1.60	1.30	22.00	1.10
CHB	28.00	234.00	13.00	11.00	3.00	1.30	20.00	1.40
PASTA	21.61	112.00	4.50	3.50	2.30	2.10	15.00	0.60
CHISA	23.04	106.00	7.20	5.10	2.10	0.90	7.40	0.70
FISA	22.00	125.00	5.40	4.30	2.00	2.00	15.20	0.40
BREAD	65.00	253.00	8.40	3.60	4.90	-	45.50	-

**Table 3:** Bioconversion parameters of BSFL reared on different FF diets. Data are expressed as means ± standard deviation from three independent biological replicates. Statistical analysis was performed with one-way ANOVA and Tukey’s *post hoc* test. Different letters indicate significant differences among groups.

Parameter	PIZZA	CHB	PASTA	CHISA	FISA	*p*-Value
Total Larval Weight (g)	32.28 ± 11.24 ^ab^	24.52 ± 8.34 ^b^	26.70 ± 4.65 ^ab^	32.56 ± 4.66 ^a^	35.21 ± 3.91 ^a^	0.0038
Larval Weight DM (g)	9.77 ± 2.34 ^ab^	7.06 ± 1.78 ^c^	7.94 ± 0.10 ^bc^	10.00 ± 1.55 ^b^	11.21 ± 0.45 ^a^	<0.0001
Larval DM (%)	30.25 ± 2.22 ^b^	28.81 ± 0.62 ^c^	29.74 ± 0.01 ^bc^	30.70 ± 0.10 ^ab^	31.85 ± 0.20 ^a^	<0.0001
Survival Rate (%)	80.18 ± 18.25 ^b^	70.25 ± 18.02 ^b^	88.25 ± 12.5 ^ab^	76.68 ± 8.46 ^b^	96.63 ± 0.40 ^a^	<0.0001
FCR ^1^	5.44 ± 2.29 ^ab^	7.34 ± 2.70 ^a^	6.19 ± 1.01 ^ab^	4.82 ± 0.94 ^b^	4.11 ± 0.59 ^b^	0.0016
FCE ^2^	0.20 ± 0.08 ^a^	0.15 ± 0.05 ^ab^	0.14 ± 0.02 ^b^	0.18 ± 0.03 ^ab^	0.17 ± 0.02 ^ab^	0.0256
FCE (DM) ^3^	0.22 ± 0.10 ^ab^	0.15 ± 0.06 ^c^	0.17 ± 0.03 ^bc^	0.22 ± 0.05 ^ab^	0.25 ± 0.00 ^a^	0.0004

^1^ Feed conversion ratio; ^2^ feed conversion efficiency; ^3^ feed conversion efficiency (in DM).

**Table 4:** Nutritional composition of BSFL reared on different FF diets (expressed as % on DM). Data are expressed as means ± standard deviation from three independent biological replicates. Statistical analysis was performed with one-way ANOVA and Tukey’s *post hoc* test. Different letters indicate significant differences among groups.

Substrate	Moisture	Crude Protein	Ether Extract	Ash	Crude Fiber
PIZZA	29.648 ± 1.179 ^a^	41.628 ± 2.564 ^ab^	20.240 ± 2.553 ^b^	4.110 ± 1.042 ^a^	4.375 ± 0.848 ^a^
CHB	26.585 ± 1.174 ^b^	43.761 ± 0.789 ^a^	25.510 ± 1.473 ^a^	2.157 ± 1.162 ^b^	1.519 ± 0.421 ^c^
PASTA	27.129 ± 1.716 ^ab^	40.076 ± 0.966 ^b^	23.510 ± 2.009 ^ab^	3.751 ± 0.628 ^ab^	2.415 ± 0.637 ^b^
CHISA	26.807 ± 2.257 ^b^	40.879 ± 2.380 ^b^	24.843 ± 3.096 ^ab^	2.701 ± 1.098 ^b^	2.682 ± 0.782 ^b^
FISA	27.311 ± 0.907 ^ab^	43.236 ± 1.543 ^a^	23.314 ± 1.518 ^ab^	2.679 ± 0.985 ^b^	2.258 ± 0.322 ^b^
*p*-value	0.0272	<0.0001	0.0026	0.0051	<0.0001

**Table 5:** Fatty acid composition (%) of BSFL reared on different FF diets. Data are expressed as means ± standard deviation from three independent biological replicates. Statistical analysis was performed with one-way ANOVA and Tukey’s *post hoc* test. *p*-value < 0.0001. Different letters indicate significant differences among groups.

Fatty Acids	PIZZA	CHB	PASTA	CHISA	FISA
**C6:0**	0.028 ± 0.008 ^a^	0.02 ± 0.002 ^b^	0.000 ± 0.000 ^c^	0.000 ± 0.000 ^c^	0.000 ± 0.000 ^c^
**C8:0**	0.049 ± 0.003 ^a^	0.076 ± 0.041 ^a^	0.000 ± 0.000 ^b^	0.000 ± 0.000 ^b^	0.000 ± 0.000 ^b^
**C10:0**	0.708 ± 0.043 ^a^	0.653 ± 0.063 ^a^	0.392 ± 0.057 ^b^	0.217 ± 0.176 ^b^	0.623 ± 0.095 ^a^
**C12:0**	25.313 ± 0.836 ^c^	31.267 ± 1.668 ^b^	34.562 ± 0.726 ^a^	24.565 ± 0.358 ^c^	33.84 ± 1.955 ^ab^
**C14:0**	9.274 ± 0.542 ^ab^	8.318 ± 0.452 ^b^	9.661 ± 1.095 ^ab^	5.51 ± 0.345 ^c^	9.811 ± 0.500 ^a^
**C14:1**	0.302 ± 0.033 ^a^	0.183 ± 0.012 ^b^	0.120 ± 0.025 ^c^	0.114 ± 0.011 ^c^	0.036 ± 0.005 ^d^
**C15:0**	0.262 ± 0.032 ^a^	0.067 ± 0.018 ^b^	0.031 ± 0.023 ^b^	0.000 ± 0.000 ^c^	0.064 ± 0.034 ^b^
**C16:0**	24.45 ± 0.839 ^a^	19.965 ± 0.888 ^b^	18.449 ± 0.576 ^c^	13.942 ± 0.299 ^d^	18.578 ± 0.758 ^c^
**C16:1**	3.085 ± 0.302 ^b^	4.418 ± 0.374 ^a^	3.461 ± 0.209 ^b^	2.076 ± 0.187 ^c^	4.445 ± 0.268 ^a^
**C17:0**	0.081 ± 0.015 ^b^	0.07 ± 0.022 ^c^	0.000 ± 0.000 ^d^	0.081 ± 0.002 ^b^	0.202 ± 0.078 ^a^
**C17:1**	0.000 ± 0.000 ^c^	0.035 ± 0.003 ^b^	0.000 ± 0.000 ^c^	0.000 ± 0.000 ^c^	0.045 ± 0.004 ^a^
**C18:1 cis 6**	0.000 ± 0.000 ^b^	0.000 ± 0.000 ^b^	0.000 ± 0.000 ^b^	0.000 ± 0.000 ^b^	0.051 ± 0.004 ^a^
**C18:0**	5.086 ± 0.579 ^a^	4.429 ± 0.323 ^a^	3.476 ± 0.279 ^b^	2.922 ± 0.071 ^c^	3.641 ± 1.057 ^ab^
**C18:1 trans 9**	0.269 ± 0.017 ^b^	0.287 ± 0.052 ^b^	0.287 ± 0.028 ^b^	0.337 ± 0.038 ^a^	0.256 ± 0.101 ^ab^
**C18:1 cis 9**	20.462 ± 0.907 ^b^	21.151 ± 1.094 ^b^	20.788 ± 1.124 ^b^	29.235 ± 0.65 ^a^	14.15 ± 0.634 ^c^
**C18:1 cis 10**	0.136 ± 0.038 ^a^	0.106 ± 0.014 ^a^	0.036 ± 0.005 ^b^	0.000 ± 0.000 ^c^	0.123 ± 0.024 ^a^
**C18:1 cis 11**	0.000 ± 0.000 ^b^	0.000 ± 0.000 ^b^	0.000 ± 0.000 ^b^	0.000 ± 0.000 ^b^	0.158 ± 0.049
**C18:2 cis ω6**	7.002 ± 0.473 ^b^	5.026 ± 1.317 ^cd^	4.708 ± 0.374 ^c^	12.233 ± 0.512 ^a^	3.353 ± 0.477 ^d^
**C20:0**	0.128 ± 0.008 ^c^	0.151 ± 0.036 ^bc^	0.128 ± 0.011 ^c^	0.194 ± 0.03 ^a^	0.193 ± 0.059 ^ab^
**C20:1**	0.073 ± 0.009 ^b^	0.098 ± 0.007 ^a^	0.051 ± 0.013 ^c^	0.111 ± 0.026 ^a^	0.000 ± 0.000 ^d^
**C18:3 ω3 (ALA)**	0.281 ± 0.02 ^d^	1.067 ± 0.499 ^c^	1.139 ± 0.095 ^c^	4.449 ± 0.361 ^a^	2.091 ± 0.418 ^b^
**C18:2 cis9trans11**	0.298 ± 0.053 ^b^	0.224 ± 0.017 ^c^	0.268 ± 0.046 ^b^	0.0 ± 0.0 ^d^	1.398 ± 0.268 ^a^
**C18:2trans10cis12**	0.000 ± 0.000 ^b^	0.000 ± 0.000 ^b^	0.000 ± 0.000 ^b^	0.000 ± 0.000 ^b^	0.048 ± 0.005 ^a^
**C22:0**	0.045 ± 0.01 ^c^	0.028 ± 0.008 ^d^	0.044 ± 0.005 ^c^	0.145 ± 0.006 ^a^	0.064 ± 0.004 ^b^
**C20:3 ω6**	0.000 ± 0.000 ^b^	0.000 ± 0.000 ^b^	0.000 ± 0.000 ^b^	0.000 ± 0.000 ^b^	1.552 ± 0.259 ^a^
**C22:1**	0.030 ± 0.002 ^b^	0.035 ± 0.012 ^a^	0.023 ± 0.004 ^c^	0.000 ± 0.000 ^d^	0.088 ± 0.026 ^a^
**C20:3 ω3**	0.000 ± 0.000 ^b^	0.048 ± 0.003 ^a^	0.000 ± 0.000 ^b^	0.000 ± 0.000 ^b^	0.055 ± 0.026 ^a^
**C20:4 ω6 (AA)**	0.057 ± 0.005 ^c^	0.072 ± 0.018 ^b^	0.055 ± 0.003 ^c^	0.294 ± 0.089 ^a^	0.089 ± 0.075 ^bc^
**C22:2 ω6**	0.000 ± 0.000 ^b^	0.000 ± 0.000 ^b^	0.000 ± 0.000 ^b^	0.000 ± 0.000 ^b^	0.132 ± 0.011 ^a^
**C24:0**	0.058 ± 0.004 ^c^	0.034 ± 0.002 ^d^	0.030 ± 0.003 ^d^	0.116 ± 0.01 ^b^	1.945 ± 0.645 ^a^
**C20:5 ω3 (EPA)**	0.000 ± 0.000 ^c^	0.000 ± 0.000 ^c^	0.000 ± 0.000 ^c^	0.086 ± 0.008 ^b^	0.333 ± 0.107 ^a^
**C24:1**	0.000 ± 0.000 ^b^	0.000 ± 0.000 ^b^	0.000 ± 0.000 ^b^	0.053 ± 0.004 ^a^	0.053 ± 0.02 ^a^
**C22:4 ω6**	0.000 ± 0.000 ^c^	0.000 ± 0.000 ^c^	0.000 ± 0.000 ^c^	0.086 ± 0.007 ^a^	0.052 ± 0.013 ^b^
**C22:5 ω3 (DPA)**	0.000 ± 0.000 ^b^	0.000 ± 0.000 ^b^	0.000 ± 0.000 ^b^	0.000 ± 0.000 ^b^	0.102 ± 0.025 ^a^
**C22:6 ω3 (DHA)**	0.000 ± 0.000 ^b^	0.000 ± 0.000 ^b^	0.000 ± 0.000 ^b^	0.000 ± 0.000 ^b^	0.707 ± 0.166 ^a^
**SFA**	67.704 ± 0.978 ^b^	66.943 ± 1.95 ^b^	68.598 ± 1.025 ^ab^	50.958 ± 0.497 ^c^	70.229 ± 0.777 ^a^
**MUFA**	24.357 ± 0.648 ^c^	26.225 ± 0.716 ^ab^	24.766 ± 1.107 ^bc^	31.926 ± 0.76 ^a^	19.278 ± 0.582 ^d^
**PUFA**	7.638 ± 0.497 ^c^	6.256 ± 1.654 ^cd^	6.169 ± 0.376 ^d^	17.149 ± 0.849 ^a^	9.824 ± 0.734 ^b^
**Omega-6**	7.060 ± 0.475 ^b^	5.098 ± 1.3 ^c^	4.763 ± 0.370 ^c^	12.613 ± 0.527 ^a^	5.112 ± 0.532 ^c^
**Omega-3**	0.281 ± 0.02 ^d^	1.083 ± 0.475 ^c^	1.139 ± 0.095 ^c^	4.535 ± 0.369 ^a^	3.289 ± 0.268 ^b^
**CLA**	0.298 ± 0.053 ^b^	0.075 ± 0.116 ^c^	0.268 ± 0.046 ^b^	0.000 ± 0.000 ^d^	1.422 ± 0.247 ^a^
**PUFA/SFA**	0.113 ± 0.009 ^c^	0.094 ± 0.027 ^c^	0.090 ± 0.006 ^c^	0.337 ± 0.019 ^a^	0.140 ± 0.012 ^b^
**Omega-6/omega-3**	25.278 ± 2.93 ^a^	5.247 ± 1.533 ^b^	4.21 ± 0.548 ^b^	2.789 ± 0.146 ^c^	1.564 ± 0.218 ^d^
**LA/ALA**	25.071 ± 2.902 ^a^	5.403 ± 1.838 ^b^	4.162 ± 0.547 ^b^	2.758 ± 0.156 ^c^	1.675 ± 0.491 ^d^
**AA/EPA**	0.000 ± 0.000 ^c^	0.000 ± 0.000 ^c^	0.000 ± 0.000 ^c^	3.386 ± 0.906 ^a^	0.227 ± 0.156 ^b^

**Table 6:** Mineral profile of BSFL reared on different FF diets. Data are expressed as means ± standard deviation from three independent biological replicates. Statistical analysis was performed with one-way ANOVA and Tukey’s *post hoc* test. *p*-value < 0.0001, except for K (*p*-value = 0.0002). Different letters indicate significant differences among groups.

Substrate	Ca mg/100 g	P mg/100 g	Mg mg/100 g	Zn mg/100 g	Fe mg/100 g
PIZZA	240.365 ± 25.555 ^a^	205.738 ± 9.096 ^b^	36.348 ± 4.050 ^b^	2.043 ± 0.177 ^d^	2.323 ± 0.259 ^b^
CHB	124.648 ± 17.092 ^b^	211.208 ± 27.867 ^b^	33.124 ± 4.460 ^b^	5.519 ± 0.506 ^a^	2.441 ± 0.489 ^b^
PASTA	121.019 ± 18.649 ^b^	198.188 ± 32.961 ^b^	62.860 ± 6.584 ^a^	0.724 ± 0.191 ^e^	2.569 ± 0.345 ^b^
CHISA	109.548 ± 52.755 ^ab^	95.091 ± 20.609 ^c^	36.442 ± 3.201 ^b^	3.376 ± 0.458 ^c^	2.076 ± 1.020 ^b^
FISA	215.410 ± 59.094 ^a^	311.635 ± 31.043 ^a^	56.677 ± 7.287 ^a^	4.668 ± 0.731 ^b^	11.406 ± 3.098 ^a^
**Substrate**	**K mg/100 g**	**Na mg/100 g**	**Cu mg/100 g**	**Mn mg/100 g**	**Se mg/100 g**
PIZZA	269.163 ± 58.401 ^c^	587.950 ± 61.779 ^b^	0.073 ± 0.033 ^c^	0.450 ± 0.063 ^a^	21.880 ± 2.234 ^c^
CHB	337.875 ± 44.686 ^b^	572.213 ± 28.318 ^b^	0.580 ± 0.526 ^b^	0.049 ± 0.008 ^c^	3.999 ± 0.415 ^d^
PASTA	354.748 ± 49.103 ^b^	95.693 ± 17.151 ^c^	0.074 ± 0.050 ^c^	0.185 ± 0.029 ^b^	1.170 ± 0.619 ^d^
CHISA	346.333 ± 54.327 ^b^	555.358 ± 164.698 ^b^	0.244 ± 0.363 ^bc^	0.180 ± 0.124 ^b^	27.809 ± 3.467 ^b^
FISA	438.700 ± 91.724 ^a^	1378.256 ± 223.487 ^a^	1.243 ± 0.319 ^a^	0.033 ± 0.012 ^c^	48.948 ± 10.274 ^a^

**Table 7:** Bioaccumulation factors (BAF) for arsenic (As), mercury (Hg), lead (Pb), and cadmium (Cd) in different substrates and corresponding larvae. Data are expressed as means ± standard deviation from three independent biological replicates.

Substrate	Heavy Metal	Concentration in Substrate (μg/100 g)	Concentration in Larvae (μg/100 g)	BAF
Pizza with salami	As	0.000 ± 0.000	0.043 ± 0.005	4.3
	Hg	0.000 ± 0.000	0.000 ± 0.000	-
	Pb	3.220 ± 0.563	4.678 ± 0.478	1.5
	Cd	0.020 ± 0.003	0.330 ± 0.065	16.5
Cheeseburger	As	0.015 ± 0.007	0.034 ± 0.013	2.3
	Hg	0.000 ± 0.000	0.000 ± 0.000	-
	Pb	2.705 ± 0.714	3.487 ± 0.653	1.3
	Cd	0.195 ± 0.049	0.408 ± 0.329	2.1
Pasta Bolognese	As	0.010 ± 0.001	0.027 ± 0.013	2.7
	Hg	0.000 ± 0.000	0.000 ± 0.000	-
	Pb	0.020 ± 0.002	0.050 ± 0.028	2.5
	Cd	0.010 ± 0.001	0.187 ± 0.009	18.7
Chicken salad	As	0.013 ± 0.006	0.015 ± 0.011	1.2
	Hg	0.000 ± 0.000	0.000 ± 0.000	-
	Pb	0.020 ± 0.01	0.015 ± 0.01	0.75
	Cd	0.013 ± 0.006	0.140 ± 0.027	10.8
Fish salad	As	0.018 ± 0.013	0.016 ± 0.009	0.89
	Hg	0.133 ± 0.028	0.143 ± 0.025	1.1
	Pb	0.013 ± 0.005	0.014 ± 0.004	1.1
	Cd	0.015 ± 0.006	0.154 ± 0.045	10.3

## Data Availability

The original contributions presented in this study are included in the article/Supplementary Materials. Further inquiries can be directed to the corresponding authors.

## Supplementary Materials

The following supporting information can be downloaded at: https://www.mdpi.com/article/10.3390/insects16050508/s1, Table S1: Heavy metals (μg/100g) of BSFL fed on former foodstuffs containing meat and fish and of initial substrate and residue. Data are presented as mean of 3 independent biological replicates. Statistical analysis was performed with one-way ANOVA (analysis of variance) and Tukey’s *post-hoc* test. Different letters indicate significant differences among groups. SEM = standard error of the mean.
