# Supplementary material for: Bioconversion of Meat and Fish-Based Former Foodstuffs by Black Soldier Fly Larvae: A Sustainable Pathway for Reducing Food Waste, Enhancing Nutrient Recovery, with a Circular Economy Approach
Source: Insects. 2025 May 9;16(5):508. doi: 10.3390/insects16050508 (PMC12112482; doi:10.3390/insects16050508)
Supplement: Supplementary file 1 [file insects-16-00508-s001.zip › insects-3532011-supplementary.pdf]

***Heavy Metals Concentration in Initial Substrates, Larvae, and Larval Frass***

| **Substrate** | **ID** | **As, µg /100g** | **Hg, µg /100g** | **Pb, µg /100g** | **Cd, µg /100g** |
|---|---|---|---|---|---|
| Pizza | Initial substrate | 0 | 0 | 3.220 ± 0.563$^{a}$ | 0.020 ± 0.003$^{b}$ |
| Chb | Initial substrate | 0.015 ± 0.007$^{a}$ | 0 | 2.705 ± 0.714$^{a}$ | 0.195 ± 0.049$^{a}$ |
| Pasta | Initial substrate | 0.010 ± 0.001$^{a}$ | 0 | 0.020 ± 0.002$^{b}$ | 0.010 ± 0.001$^{c}$ |
| Chi salad | Initial substrate | 0.013 ± 0.006$^{a}$ | 0 | 0.020 ± 0.010$^{b}$ | 0.013 ± 0.006$^{bc}$ |
| Fish salad | Initial substrate | 0.018 ± 0.013$^{a}$ | 0.133 ± 0.028$^{a}$ | 0.013 ± 0.005$^{b}$ | 0.015 ± 0.006$^{bc}$ |
| **P-value** | | 0.0264 | <0.0001 | <0.0001 | <0.0001 |

| **Substrate** | **ID** | **As, µg/100g** | **Hg, µg/100g** | **Pb, µg/100g** | **Cd, µg/100g** |
|---|---|---|---|---|---|
| Pizza | Larvae | 0.043 ± 0.005$^{a}$ | 0 | 4.678 ± 0.478$^{a}$ | 0.330 ± 0.065$^{a}$ |
| Chb | Larvae | 0.034 ± 0.013$^{ab}$ | 0 | 3.487 ± 0.653$^{b}$ | 0.408 ± 0.329$^{a}$ |
| Pasta | Larvae | 0.027 ± 0.013$^{ab}$ | 0 | 0.050 ± 0.028$^{c}$ | 0.187 ± 0.009$^{a}$ |
| Chi salad | Larvae | 0.015 ± 0.011$^{b}$ | 0 | 0.015 ± 0.010$^{cd}$ | 0.140 ± 0.027$^{a}$ |
| Fish | Larvae | 0.016 ± 0.009$^{b}$ | 0.143 ± 0.025$^{a}$ | 0.014 ± 0.004$^{d}$ | 0.154 ± 0.045$^{a}$ |
| **P-value** | | 0.0089 | <0.001 | <0.001 | 0.0915 |

| **Substrate** | **ID** | **As, µg/100g** | **Hg, µg/100g** | **Pb, µg/100g** | **Cd, µg/100g** |
|---|---|---|---|---|---|
| Pizza | Residue | 0.040 ± 0.006$^{a}$ | 0 | 3.954 ± 0.703$^{a}$ | 0.180 ± 0.043$^{b}$ |
| Chb | Residue | 0.021 ± 0.020$^{ab}$ | 0 | 2.786 ± 1.296$^{a}$ | 0.473 ± 0.396$^{ab}$ |
| Pasta | Residue | 0.020 ± 0.010$^{b}$ | 0 | 0.030 ± 0.028$^{b}$ | 0.190 ± 0.081$^{b}$ |
| Chi salad | Residue | 0.012 ± 0.011$^{b}$ | 0 | 0.006 ± 0.005$^{b}$ | 0.047 ± 0.042$^{c}$ |
| Fish | Residue | 0.011 ± 0.010$^{b}$ | 0.096 ± 0.023$^{a}$ | 0.025 ± 0.021$^{b}$ | 0.069 ± 0.023$^{c}$ |
| **P-value** | | 0.0304 | <0.001 | <0.001 | 0.0348 |

**Supplementary Table S1**: Heavy metals (µg/100g) of BSFL fed on former foodstuffs containing meat and fish and of initial substrate and residue. Data are presented as mean of 3 independent biological replicates. Statistical analysis was performed with one-way ANOVA (analysis of variance) and Tukey's *post-hoc* test. Different letters indicate significant differences among groups. SEM = standard error of the mean.
